# The impact of laparoscopic versus open inguinal hernia repair for inguinal hernia treatment: A retrospective cohort study

**DOI:** 10.1002/hsr2.1194

**Published:** 2023-04-11

**Authors:** Yong Zhao, Zipeng Xu, Tao Wang, Dingxing Zhou, Neng Tang, Shuo Zhang, Chaobo Chen

**Affiliations:** ^1^ Department of General Surgery Wuxi Rehabilitation Hospital Wuxi China; ^2^ Department of General Surgery Xishan People's Hospital of Wuxi City Wuxi China; ^3^ Department of Emergency Surgery Wuxi Second Hospital of Traditional Chinese Medicine Wuxi China; ^4^ Department of Hepatic‐Biliary‐Pancreatic Surgery the Affiliated Drum Tower Hospital of Nanjing University Medical school Nanjing China; ^5^ Department of Immunology, Ophthalmology & ORL Complutense University School of Medicine Madrid Spain

**Keywords:** inguinal hernia, laparoscopy, minimally invasive surgery, retrospective study

## Abstract

**Objectives:**

Although laparoscopic inguinal hernia repair (LIHR) has been widely accepted for treating inguinal hernia, the procedure remains very technical and challenging. The present study aimed to assess the effect of LIHR in relation to operation time, intraoperative hemorrhage and postoperative hospitalization.

**Methods:**

A total of 503 patients with inguinal hernia admitted at the Wuxi Rehabilitation Hospital between June 2019 and July 2021 were included in this retrospective cohort study. Binary logistic and linear regressions were used for categorical and continuous outcomes, respectively. The learning curve was drawn by cumulative sum analysis.

**Results:**

Multivariate logistic regression analysis identified LIHR as an independent factor associated with prolonging operation time (odd ratio [OR] = 1.750, 95% confidence interval [CI]: 1.215−2.520, *p* = 0.003) and decreasing intraoperative hemorrhage levels (OR = 0.079, 95 CI: 0.044−0.142, *p* < 0.001). Multivariate linear regression identified LIHR (Coefficient = −0.702, 95% CI: [−1.050] to [−0.354], *p* < 0.001) as an independent factor for shortening postoperative hospitalization time. After learning curve, LIHR (OR = 1.409, 95% CI: 0.948 to 2.094, *p* = 0.090) no longer resulted as a risk factor prolonging operation time.

**Conclusions:**

LIHR is an important independent predictive factor for decreasing intraoperative hemorrhage levels and shortening postoperative hospitalization time. Additionally, LIHR does not prolong operation time after the learning curve.

## 1 INTRODUCTION

A primary inguinal hernia is a common abdominal surgical disease that is usually caused by defects in the abdominal wall in the groin area. Epidemiologically, family history, male sex, advanced age, low body mass index, systemic connective tissue disease, prostate surgery, and history of radiation therapy are all risk factors for inguinal hernia.^1^ In women, inguinal hernias have also been associated with higher height, chronic cough, and more. However, there is still no evidence‐based medical evidence to support a relationship between smoking and/or drinking alcohol and hernias.^2^ It has been reported that the frequency of inguinal hernia repair increases with age, that is, 0.25%−4.2% in 18−80 year‐olds.^3^ According to the anatomical relationship of the Hessel Bach triangle, an inguinal hernia can be divided into the direct inguinal hernia, indirect inguinal hernia and femoral hernia, where approximately 96% are inguinal hernias, 20% of them are bilateral.^4^ Inguinal hernias are more common in men, while femoral hernias are more common in women.^5^ People with inguinal hernias may report a bulge or mass in the groin that becomes bigger over time. Stretching or tearing of the hernia defect site or surrounding tissue may cause pain or fainting; yet, approximately 1/3 of patients tend to be asymptomatic.^6^ Importantly, acute severe abdominal pain suggests that the hernia may be incarcerated, requiring urgent surgical treatment.^3^ Generally, once an inguinal hernia is formed, surgery is the most effective treatment. Inguinal hernia repair, including minimally invasive surgery and traditional open surgery, is the most common procedure. Although laparoscopic inguinal hernia repair (LIHR) has been accepted worldwide for the treatment of inguinal hernia, there is still some controversy between LIHR and open inguinal hernia repair (OIHR).^7^, ^8^ Furthermore, the promotion of LIHR technology is based on surgeons overcoming the learning curve, otherwise patients may encounter increased cost, longer operating time, higher recurrence, and complication rates.^9^, ^10^

Since the early 1990s, when LIHR was first reported,^11^ the LIHR has become a routine procedure due to the rapid development of minimally invasive surgical technology and the increasing precision of surgical instruments and equipment. At present, an increasing number of surgeons advocate LIHR as an alternative to OIHR, although the previous alternative to OIHR was the open anterior approach, which was extensively used worldwide for the repair of the majority of hernias.^12^

For a long time, recurrence has been considered an important outcome measure of the quality of inguinal hernia repair. Recurrence rates after OIHR were reported to be as high as 6.3% versus 6.5% after LIHR.^13^ However, it was also reported that LIHR has advantages compared to OIHR approach in view of postoperative pain, time needed to return to work, and chronic inguinal pain.^14^ Even so, its potential has not been fully used for the benefit of the patients. Although unilateral inguinal hernias may be controversial, the benefit of LIHR over open repair is well‐established in patients with bilateral or recurrent inguinal hernias, and current guidelines recommend minimally invasive surgical repair for such cases.^15^ Furthermore, the use of LIHR is not age‐restricted, so elderly patients can also benefit from this approach, as previously reported.^16^ Still, patients with inguinal hernia tend to have individual differences, so preoperative evaluation of age, operation time, hernia sac diameter, intraoperative hemorrhage, postoperative hospitalization, postoperative complications, and other factors may also influence the surgeon's choice of a surgical method for treating the patients. Therefore, choosing a more appropriate surgical method for such patients is of great importance.

In China, LIHR has successfully treated patients with inguinal hernia over the last 10 years in many medical centers, achieving good feedback.^17^ However, the choice of treatment techniques for an inguinal hernia as well as different concepts may vary across different regions and medical centers, thus failing to achieve standardization of surgical treatment of inguinal hernia. Herein, we retrospectively summarized the experience and impact of LIHR versus OIHR on the treatment of inguinal hernia in our medical center, which may further benefit patients in future treatment.

## 2 METHODS

### 2.1 Study design and patients

Patients with inguinal hernia admitted at the Wuxi Rehabilitation Hospital between June 2019 and July 2021 were included in this retrospective cohort study.

Inclusion criteria were the following: (1) those who underwent either LIHR or OIHR; (2) aged 18−75 years; (3) with unilateral inguinal hernia.

Exclusion criteria were the following: (1) patients undergoing surgery who converted from LIHR to OIHR; (2) with complexed hernias (such as, obstructive or/and strangulated hernias); (3) uncorrectable coagulation disorders; (4) pregnant women; (5) recurrent inguinal hernia.

The study complied with the Declaration of Helsinki and was approved by the Ethics Committee of the Wuxi Rehabilitation Hospital (No. wxkf20220618). The requirement for informed consent was waived.

### 2.2 Surgical technique

All surgeries were completed by the same surgical team. Surgical procedures for LIHR and OIHR were performed as previously published.^18^, ^19^, ^20^ In this study, LIHR was performed as transabdominal preperitoneal procedure (TAPP), whilst OIHR was performed as Lichtenstein techniques.

### 2.3 Data collection

Data collection and follow‐up were carried out for both LIHR and OIHR groups. Following data were included: age, medical history, hernia sac diameter, gender (M/F), type of hernia (Indirect/direct hernia), operation time, intraoperative hemorrhage, and postoperative hospitalization.

### 2.4 Statistical analysis

SPSS 22.0 (IBM) was used for data analysis. The continuous data were expressed as means ± standard deviations and analyzed via Student's *t*‐test. Categorical data were presented as frequencies and scores and were analyzed using *χ* ^2^ test and Fisher's exact test. Non‐normally distributed variables were presented as medians with interquartile ranges and were tested using the Mann–Whitney *U* test. Binary logistic and linear regressions were used for categorical and continuous outcomes, respectively. To perform binary logistic regression, we categorized study participants into short operation time group and long operation time group based on median operation time in our study population. Meanwhile, patients were stratified into groups with less bleeding and more bleeding based on median intraoperative hemorrhage levels. The learning curve was drawn by cumulative sum (CUSUM) analysis. A *p* < 0.05 was considered statistically significant.

## 3 RESULTS

### 3.1 Characteristics of the patients

**Figure 1:**
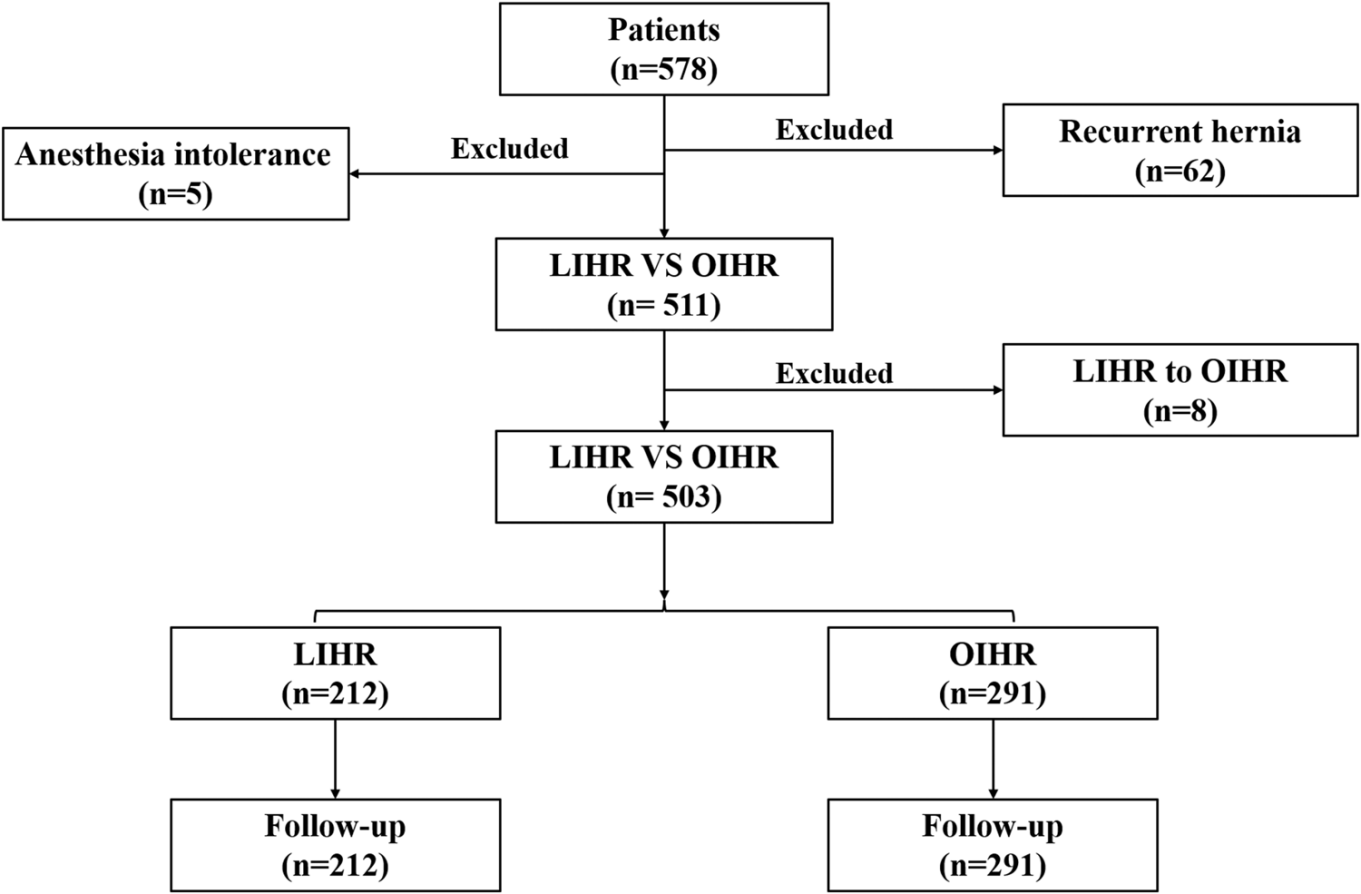
Detailed surgery flow diagram.

A total of 578 patients with primary unilateral inguinal hernias diagnosed as inguinal hernia at Wuxi Rehabilitation Hospital were initially identified. Among 578 patients, 5 were excluded due to anesthesia intolerance, and 62 were excluded due to recurrent hernia combined with second surgery treatment. A total of 220 patients were treated with LIHR; 8 were excluded because they converted from LIHR to OIHR. Finally, 503 patients, 212 treated with LIHR and 291 treated with OIHR, were included in this study (Figure 1).

Significant differences were observed in age, medical history, Type of hernia (Indirect or direct hernia) and ASA Grade (I/II/III) (all *p* < 0.01, Table 1) between OIHR and LIHR groups, while there were no differences in gender and hernia sac diameter (all *p* > 0.05, Table 1).

**Table 1:** Characteristic and clinical features of patients.

Variables	LIHR	OIHR	*p* Value
Age	59 (50−67)	68 (58−76)	*<0.001*
Medical history, median (IQR), Month	5 (1−12)	12 (2−36)	*0.001*
Hernia sac diameter, median (IQR), mm	40 (40−60)	46 (40−60)	0.140
Gender (F/M)	7/205	15/276	0.316
Type of hernia (direct/indirect hernia)	46/166	41/250	*0.026*
Operation time, median (IQR), min	85 (70−95)	75 (64−90)	*<0.001*
Intraoperative hemorrhage, median (IQR), ml	10 (10−10)	20 (10−20)	*<0.001*
Postoperative hospitalization, median (IQR), day	2.5 (2−4)	4 (2.5−5)	*<0.001*
ASA Grade (I/II/III)	158/54/0	159/124/8	*<0.001*

*Note*: Italic values are statistically significant at *p* < 0.05.

Abbreviations: ASA, American Society of Anesthesiologists; IQR, interquartile ranges; LIHR, laparoscopic inguinal hernia repair; OIHR, open inguinal hernia repair.

### 3.2 Potential effect factors associated with operative time

As shown in Table 1, the operation time of the LIHR group was longer than the OIHR group (85 [70−95] vs. 75 [64−90], *p* < 0.001). However, there were some significantly different variables between the two groups. To identify potential factors associated with operation time, we performed binary logistic regression, which revealed that LIHR and hernia sac diameter was associated with longer operation time (Table 2). In addition, multivariate logistic regression indicated that LIHR (OR = 1.750, 95% CI: 1.215−2.520, *p* = 0.003) and hernia sac diameter (OR = 1.013, 95%: 1.004−1.021, *p* = 0.003) were independent risk factors for prolonging operation time (Table 2).

**Table 2:** Univariable and multivariable analysis for *Operation time*.

Items	Univariate logistic regression			Multivariable logistic regression		
	OR	95% CI	*p* Value	OR	95% CI	*p* Value
Surgical method (LIHR vs. OIHR)	1.621	1.133−2.320	*0.008*	1.750	1.215−2.520	*0.003*
Gender (F/M)	1.270	0.533−3.026	0.590			
Type of hernia (direct/indirect hernia)	0.822	0.517−1.305	0.405			
Medical history (month)	1.002	1.000−1.004	0.055			
Hernia sac diameter (mm)	1.011	1.003−1.020	*0.009*	1.013	1.004‐1.021	*0.003*
Age (year)	0.988	0.976−1.001	0.068			
ASA grade	0.794	0.566−1.114	0.182			

*Note*: Italic values are statistically significant at *p* < 0.05.

Abbreviations: CI, confidence interval; OR, odd ratio; LIHR, laparoscopic inguinal hernia repair; OIHR, open inguinal hernia repair.

### 3.3 Potential factors affected intraoperative hemorrhage levels

In this study, the LIHR group had less intraoperative hemorrhage than OIHR group (10 [10−10] vs. 20 [10−20], *p* < 0.001) (Table 1). Univariable analysis showed that hernia sac diameter and age were positively related to intraoperative hemorrhage levels while LIHR was inversely associated with intraoperative hemorrhage levels (Table 3). The multivariable regression analysis, which was conducted to identify independent predictors affecting intraoperative hemorrhage, revealed LIHR (OR = 0.079, 95 CI: 0.044−0.142, *p* < 0.001) as an independent factor for decreasing intraoperative hemorrhage levels (Table 3).

**Table 3:** Univariable and multivariable analysis for *Intraoperative hemorrhage* in all patients.

Items	Univariate logistic regression			Multivariable logistic regression		
	OR	95% CI	*p* Value	OR	95% CI	*p* Value
Surgical method (LIHR vs. OIHR)	0.073	0.041−0.129	*<0.001*	0.079	0.044−0.142	*<0.001*
Gender (F/M)	1.456	0.609−3.480	0.398			
Type of hernia (direct/indirect hernia)	1.108	0.680−1.804	0.681			
Medical history (Month)	1.001	0.999−1.003	0.175			
Hernia sac diameter (mm)	1.011	1.003−1.019	*0.008*	1.007	0.998−1.015	0.132
Age (year)	1.028	1.013−1.043	*<0.001*	1.007	0.992−1.023	0.349
ASA grade	1.380	0.969−1.967	0.075			

*Note*: Italic values are statistically significant at *p* < 0.05.

Abbreviations: ASA, American Society of Anesthesiologists; IQR, interquartile ranges; LIHR, laparoscopic inguinal hernia repair; OIHR, open inguinal hernia repair.

### 3.4 Potential influence factors related to postoperative hospitalization time

Herein, we determined that postoperative hospitalization of the LIHR group was shorter than the OIHR group (2.5 [2−4] versus 4 [2.5−5], *p* < 0.001) (Table 4). To identify the best predictors related with postoperative hospitalization time, we performed a linear regression analysis on all elements. Our results suggested that medical history and age prolonged the time of postoperative hospitalization while LIHR approach shorten inpatient time after operation. Meanwhile, multivariate linear regression showed that LIHR is an independent factor for shortening postoperative hospitalization time (Coefficient = −0.702, 95%CI: [−1.050] to [−0.354], *p* < 0.001).

**Table 4:** Univariable and multivariable analysis for *postoperative hospitalization* in all patients.

Items	Univariate linear regression			Multivariable linear regression		
	Coefficient	95% CI	*p* Value	Coefficient	95% CI	*p* Value
Surgical method (LIHR vs. OIHR)	−0.805	(−1.136) to (−0.474)	*<0.001*	−0.702	(−1.050) to (−0.354)	*<0.001*
Gender (F/M)	0.042	(−0.775) to 0.859	0.920			
Type of hernia (direct/indirect hernia)	−0.152	(−0.593) to 0.29	0.500			
Medical history (month)	0.002	0.000−0.003	*0.030*	0.001	0.000−0.003	0.080
Hernia sac diameter (mm)	−0.003	(−0.01) to 0.005	0.491			
Age (year)	0.015	0.003−0.026	*0.015*	0.008	(−0.004) to 0.020	0.184
ASA grade	1.380	0.969−1.967	0.075			

*Note*: Italic values are statistically significant at *p* < 0.05.

### 3.5 Potential factors influenced operative time and intraoperative hemorrhage levels after the learning curve

**Figure 2:**
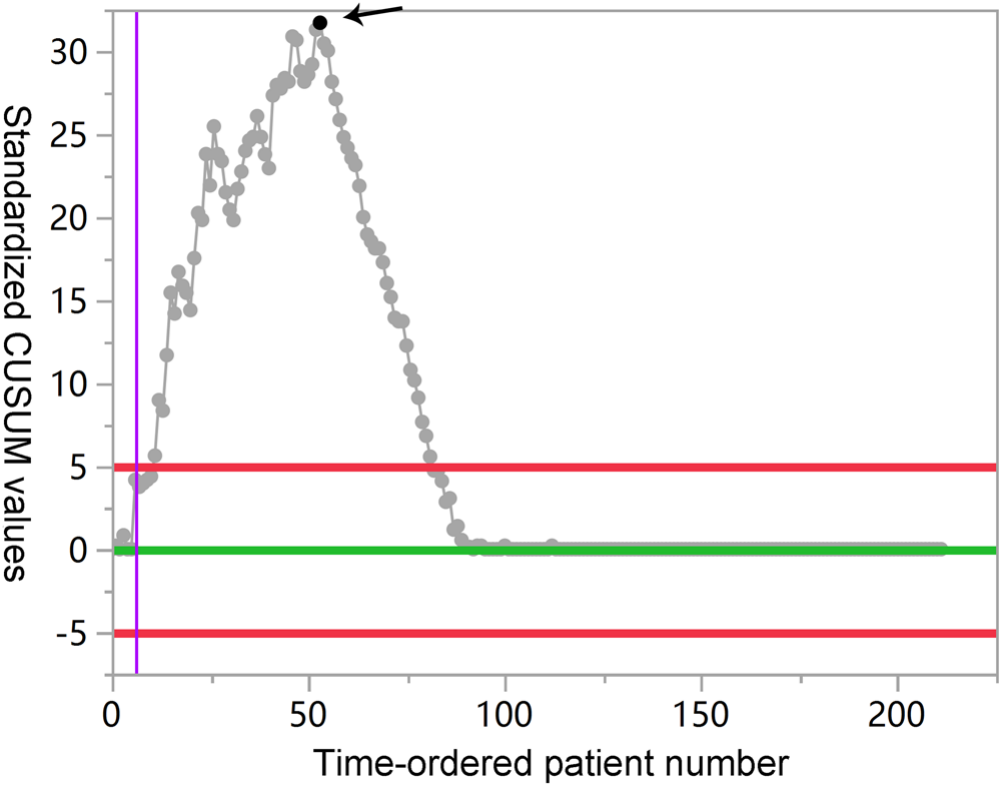
CUSUM curve based on operation time for LIHR. The target value was set as the average operation time. The black arrows indicated the 54th patient. LIHR, laparoscopic inguinal hernia repair.

It is well known that the proficiency of operation has an impact on operative time and intraoperative hemorrhage levels.^21^ Hence, CUSUM analysis was performed to plot learning curve for LIHR. As shown in Figure 2, during the 54th case, the trend of CUSUM analysis increased and then decreased. Therefore, the learning curve of LIHR was defined as 54th cases. Interestingly, LIHR was still an independent factor for decreasing intraoperative hemorrhage levels (OR = 0.047, 95 CI%: 0.021−0.105, *p* < 0.001) while not the independent risk factor prolonging operation time after learning curve (OR = 1.409, 95% CI: 0.948−2.094, *p* = 0.090) (Tables 5 and 6). These results revealed that the method of operation was no longer a risk factor to the operation time after learning curve.

**Table 5:** Multivariable analysis for *Operation time and Intraoperative hemorrhage* in all patients after the learning curve.

Items	Operation time			Intraoperative hemorrhage		
	OR	95% CI	*p* Value	OR	95% CI	*p* Value
Surgical method (LIHR vs. OIHR)	1.409	0.948−2.094	0.090	0.047	0.021−0.105	*<0.001*
Medical history (Month)	1.001	0.999−1.003	0.155	‐	‐	‐
Hernia sac diameter (mm)	1.011	1.002−1.021	*0.021*	1.004	0.996−1.013	0.335
Age (year)	‐	‐	*‐*	1.005	0.989−1.021	0.568

*Note*: Italic values are statistically significant at *p* < 0.05.

**Table 6:** Univariable analysis for *Operation time and Intraoperative hemorrhage* in all patients after a learning curve.

Items	Operation time			Intraoperative hemorrhage		
	OR	95% CI	*p* Value	OR	95% CI	*p* Value
Surgical method (LIHR vs. OIHR)	1.271	0.862−1.874	0.227	0.044	0.020−0.098	***<0.001***
Gender (F/M)	1.321	0.545−3.200	0.538	1.434	0.591−3.481	0.426
Type of hernia (direct/indirect hernia)	0.837	0.517−1.355	0.469	1.058	0.640−1.749	0.825
Medical history (month)	1.002	1.000−1.004	***0.035***	1.001	0.999−1.003	0.211
Hernia sac diameter (mm)	1.012	1.004−1.021	***0.006***	1.009	1.001−1.017	***0.031***
Age (year)	0.989	0.977−1.002	*0.099*	1.025	1.011−1.041	***0.001***
ASA grade	0.922	0.650−1.308	0.649	1.291	0.897−1.858	0.170

*Note*: Italic values are statistically significant at *p* < 0.05.

Abbreviations: ASA, American Society of Anesthesiologists; IQR, interquartile ranges; LIHR, laparoscopic inguinal hernia repair; OIHR, open inguinal hernia repair.

## 4 DISCUSSION

Inguinal hernia is commonly managed by surgical treatment. In this study, we compared the patients who underwent either LIHR or OIHR to analyze the differences in operation time, intraoperative hemorrhage, postoperative hospitalization, and medical history. Importantly, LIHR no longer resulted as a risk factor for the operation time after the learning curve. Actually, these data may guide surgeons in choosing suitable operation methods for different patients in the later period.

Since the first LIHR,^11^ this approach has been associated with various advantages such as less trauma, reduced postoperative pain and wound infection, as well as less time needed to return to work, etc. Patients with inguinal hernia could benefit from LIHR, considering it is the minimally invasive surgery for treating inguinal hernia.^22^

Herein, we assessed the advantages and disadvantages between LIHR and OIHR. Compared with LIHR, OIHR had a shorter operation time. Statistical analysis suggested that LIHR was an independent factor for prolonged operation time in the treatment of inguinal hernia (Table 2), which was consistent with a previous study.^16^ Of note, age, surgical technique, size of inguinal hernia, and teamwork could affect the operation time, but not only in LIHR.^23^, ^24^ Therefore, we also plotted the learning curve of LIHR. Interestingly, LIHR no longer resulted as a risk factor for prolonging operation time based on the presentation of the learning curve (Figure 2). However, LIHR had great advantages in decreasing intraoperative hemorrhage levels and shortening postoperative hospitalization time compared to OIHR. Previous study has shown that the LIHR technique requires a certain learning curve for the surgeon, and patients also need to undergo general anesthesia.^25^ When surgeons overcome their learning curve (previously reported range from 50 to 250 cases,^26^ which was in line with our study of 54 cases, Figure 2), the potential advantages of LIHR, such as faster recovery, reduced pain and recurrence rates, and similar will be more effective.^9^

Even though there was no recurrence of hernias at our medical center between June 2019 and July 2021, the same surgical group completed all of the operations. Numerous studies have shown that LIHR was similar to OIHR in terms of recurrence rates^27^; however, long‐term follow‐up data are still needed. Previously, LIHR of primary inguinal hernias in women was associated with lower reoperation rates and less recurrence of femoral hernias than the OIHR approach.^13^ It has also been reported that untreated lipoma is the main cause of recurrence after laparoscopic repair, so any lipoma in the inguinal region must be clearly dissected during the LIHR.^28^ Several technical factors, such as mesh size, improper fixation, and missed hernias, are generally considered the main causes of recurrent hernias in OIHR and LIHR.^12^ In addition, insufficient medial/lateral fixation of the mesh, and omission of lipomas and hernias through mesh slits are also factors for hernia recurrence. Likewise, insufficient separation and overlap of myopectineal orifice, mesh folding, and hematoma‐induced dislocations have also been suggested as factors affecting hernia recurrence.^29^ Therefore, the LIHR approach should be implemented by experienced surgeons.^12^ To carry out the correct procedure of LIHR, the surgeons must understand the anatomical structure of the Myopectineal Orifice,^12^, ^15^ which coincides with the surgeon's learning curve.

In addition, when accounting for potential factors influencing operative time and intraoperative hemorrhage levels according to the learning curve plotted for LIHR, the method of operation no longer resulted as a risk factor to the operation time after learning curve. Accordingly, we believe that improving surgical skills and surpassing the learning curve can highlight the advantages of minimally invasive surgery in diagnosis and treatment, that is, the advantages of LIHR in the treatment of inguinal hernia patients.

### 4.1 Study limitations

The present study has some limitations, like a comparison of the treatment of unilateral inguinal hernias. In addition, more feedback from patients undergoing this procedure is needed. Also, this was a retrospective study, so large data samples and prospective studies may better characterize the association between hernia sac diameter, medical history, surgical method, operation time, intraoperative hemorrhage and length of stay. Moreover, long‐term follow‐up was not performed. Furthermore, patients were mainly from nearby of Wuxi City, Eastern China, and there was a lack of multi‐center and/or regional comparative analysis.

## 5 CONCLUSION

This study elaborated on the treatment of inguinal hernia in one medical center in Eastern China. Despite improved technology, LIHR resulted not only as a safe and feasible strategy in general, but also as an advantageous approach for treating inguinal hernia. In fact, LIHR was an important independent predictive factor for decreasing intraoperative hemorrhage levels and shortening postoperative hospitalization time. Therefore, it is very important that surgeons advance their surgical skills and cross the learning curve of LIHR, which is an important basis for the full realization of the advantages of minimally invasive diagnosis and treatment of patients with inguinal hernia.

## AUTHOR CONTRIBUTIONS

**Yong Zhao**: Data curation; visualization. **Tao Wang**: Funding acquisition; supervision; validation. **Dingxing Zhou**: Data curation; software; validation. **Neng Tang**: Investigation; validation. **Shuo Zhang**: Conceptualization; data curation; supervision. **Chaobo Chen**: Formal analysis; funding acquisition; visualization; writing—original draft; writing—review and editing.

## CONFLICT OF INTEREST STATEMENT

The authors declared no conflict of interest.

## ETHICS STATEMENT

This study was performed in line with the principles of the Declaration of Helsinki. Approval was granted by the Ethics Committee of the Wuxi Rehabilitation Hospital (No. wxkf20220618). The need for individual consent was waived by the committee.

## TRANSPARENCY STATEMENT

The lead author Chaobo Chen affirms that this manuscript is an honest, accurate, and transparent account of the study being reported; that no important aspects of the study have been omitted; and that any discrepancies from the study as planned (and, if relevant, registered) have been explained.

## Data Availability

The datasets used and/or analyzed during the current study are available from the corresponding author (Chaobo Chen) upon reasonable request. For any queries, kindly contact bobo19820106@gmail.com.
